# Safety and efficacy of PEG-rhG-CSF as primary prophylaxis for neutropenia in gastrointestinal cancer patients receiving combination chemotherapy including oral agents: a prospective, exploratory, non-randomized controlled study

**DOI:** 10.3389/fonc.2026.1764358

**Published:** 2026-04-14

**Authors:** Hui Wang, Xiaoling Zhang, Yunyi Du, Min Liu, Ning Ma, Yangjun Gao, Mei Wang, Linlin Cai, Tingting Feng, Suxia Yang, Wenqing Hu, Gehong Zhang, Jun Zhao

**Affiliations:** 1 Department of Oncology, Changzhi People’s Hospital Affiliated to Changzhi Medical College, Changzhi, Shanxi, China; 2 Department of Basic Medical Sciences, Shanxi Medical University, Taiyuan, Shanxi, China; 3 Department of Gastrointestinal Surgery, Changzhi People’s Hospital Affiliated to Changzhi Medical College, Changzhi, Shanxi, China; 4 Department of Comprehensive Oncology, First Hospital of Shanxi Medical University, Taiyuan, Shanxi, China

**Keywords:** chemotherapy-induced neutropenia, gastrointestinal neoplasms, oral chemotherapy agents, PEG-rhG-CSF, primary prophylaxis

## Abstract

**Purpose:**

The safety and efficacy of pegylated recombinant human granulocyte colony-stimulating factor (PEG-rhG-CSF) for prevention of chemotherapy-induced neutropenia (CIN) in patients undergoing oral chemotherapy remain unclear. This study aimed to investigate the safety and efficacy of PEG-rhG-CSF as primary prophylaxis for CIN in gastrointestinal (GI) cancer patients receiving combination chemotherapy regimens that include oral chemotherapy agents.

**Methods:**

This is a prospective, single-center, open-label, exploratory, non-randomized controlled study. GI cancer patients was treated with intravenous oxaliplatin (130mg/m^2^ on day 1) combined with either oral capecitabine (1000mg/m^2^) or S−1 (an oral fluoropyrimidine combination of tegafur, gimeracil, and oteracil potassium; 40–60 mg) administered twice daily on days 1–14 of a 3−week cycle. The treatment group received subcutaneous injection of PEG-rhG-CSF (6mg) 24 hours after oxaliplatin, while the control group received no primary prophylaxis. The primary endpoint was safety, and secondary endpoints included the incidence of CIN.

**Results:**

Between March 2022 and January 2023, 49 patients were screened, and 43 patients who completed at least two treatment cycles were included in the final analysis (26 in treatment group and 17 in control group). The overall adverse events (AEs) did not differ statistically (93.8% vs 100.0%, *p* = 0.542). For grade ≥ 3 AEs, the incidence of neutropenia was significantly lower in the treatment group compared to the control group (3.1% vs 35.3%, *p* = 0.005). No significant differences were observed in the rates of grade ≥ 3 thrombocytopenia (6.3% vs 17.6%, *p* = 0.326) and leukopenia (3.1% vs 0.0%, *p* = 1.000). Grade≥2 CIN was significantly lower in the treatment group (25.0% vs. 76.5%, *p* < 0.001).

**Conclusion:**

In GI cancer patients on oral agents, primary PEG-rhG-CSF prophylaxis was well-tolerated and reduced grade ≥2 CIN, though randomized studies are needed.

**Clinical Trial Registration:**

https://www.chictr.org.cn, identifier ChiCTR2100054854.

## 1 Introduction

Gastrointestinal cancers are among the most common malignancies globally, with gastric cancer and colorectal cancer ranking third and fifth in incidence, and second and fifth in mortality, respectively, thereby representing a significant public health burden (1). Current clinical practice guidelines from the National Comprehensive Cancer Network (NCCN) (2, 3)、the European Society for Medical Oncology (ESMO) (4, 5), and the Chinese Society of Clinical Oncology (CSCO) (6, 7) recommend combination chemotherapy regimens incorporating capecitabine or S−1 (an oral fluoropyrimidine combination of tegafur, gimeracil, and oteracil potassium) as fundamental components of perioperative and advanced-stage therapeutic strategies for gastrointestinal malignancies.

However, patients receiving such regimens frequently experience chemotherapy-induced neutropenia (CIN), with an incidence as high as 54.7% (8–10). Severe CIN, particularly febrile neutropenia (FN), may precipitate infections, necessitate chemotherapy delays or dose reductions, and ultimately compromise clinical outcomes (11–13). The distinctive feature of oral chemotherapy lies in its requirement for continuous administration over a 14-day cycle. Should CIN or FN occur during this period, treatment must be interrupted until absolute neutrophil count (ANC) recover, thereby disrupting therapeutic continuity and diminishing efficacy. Consequently, effective prevention of CIN and FN is of paramount importance for this patient population.

Current guidelines recommend the prophylactic use of pegylated recombinant human granulocyte colony-stimulating factor (PEG-rhG-CSF) in patients at high risk for FN to effectively reduce the incidence of CIN and FN (14, 15). As a long-acting formulation, PEG-rhG-CSF provides sustained stimulation of neutrophil production and maturation (16). The standard regimen involves administration 24 hours after completion of chemotherapy. However, when patients require continued oral chemotherapy following PEG-rhG-CSF injection, robust prospective evidence regarding its safety and efficacy remains lacking.

Several studies have investigated this issue. In a study of neoadjuvant therapy for breast cancer, Wenzel et al. reported that the concomitant use of capecitabine and PEG−rhG−CSF was safe (17). A retrospective study on gastrointestinal malignancies reached a similar conclusion (18), however, its retrospective design may introduce selection bias and incomplete data. To address this clinical question, we designed a prospective controlled study aimed at providing more robust evidence on the safety and efficacy of primary prophylactic PEG-rhG-CSF in patients with gastrointestinal malignancies receiving regimens containing oral chemotherapeutic agents.

## 2 Methods

### 2.1 Study design and patients

This study was a prospective, single-center, open-label, exploratory, non-randomized controlled trial. The protocol was approved by the Ethics Review Committee of Chinese Registered Clinical Trial (Approval No. ChiECRCT20210557) and conducted in accordance with the principles of the Declaration of Helsinki (revised in 2013). Written informed consent was obtained from all participants. The trial was registered with the Chinese Clinical Trial Registry (Registration No. ChiCTR2100054854).

Inclusion criteria were as follows: (1) histologically or cytologically confirmed gastric or colorectal carcinoma; (2) age ≥18 years, with no sex restriction; (3) Eastern Cooperative Oncology Group (ECOG) performance status score of 0–1 and an anticipated survival of at least 3 months; (4) adequate organ function; (5) planned administration of at least two consecutive cycles of a chemotherapy regimen containing capecitabine or S−1; (6) presence of at least one high-risk factor for FN, defined as assessed at baseline prior to cycle 1: (6a) age ≥65 years with planned full-dose chemotherapy; (6b) prior chemotherapy or radiotherapy; (6c) malnutrition or nutritional risk: NRS-2002 score ≥3;(6d) hepatic dysfunction: total bilirubin >2.0 mg/dL; (6e) renal impairment: creatinine clearance ≤50 mL/min (Cockcroft–Gault); (6f) advanced disease stage: unresectable stage IV or metastatic disease (19).; (7) no prior history of FN or grade ≥3 chemotherapy-induced neutropenia during previous treatments; (8) ability to comprehend and willingness to provide written informed consent.

Exclusion criteria were as follows: (1) presence of uncontrolled infection at the time of enrollment; (2) history of other malignancies within the past five years; (3) confirmed bone marrow metastasis or hematopoietic dysfunction; (4) known hypersensitivity to PEG-rhG-CSF or other biological agents; (5) pregnancy or lactation; and (6) any other condition deemed unsuitable for participation by the investigators.

### 2.2 Treatment protocol

All patients received either the SOX regimen (oxaliplatin + S-1) or the CAPOX regimen (oxaliplatin + capecitabine), with each treatment cycle lasting 21 days. Oxaliplatin was administered intravenously at a dose of 130 mg/m² on day 1. The S-1 dose was adjusted according to body surface area (BSA) and given orally twice daily on days 1–14 as follows: BSA < 1.25 m², 40 mg per dose; 1.25 m² ≤ BSA < 1.5 m², 50 mg per dose; BSA ≥ 1.5 m², 60 mg per dose. Capecitabine was administered orally at 1,000 mg/m² twice daily on days 1–14. According to the NCCN guidelines, these regimens carry a moderate risk (10–20%) of febrile neutropenia (15).

In the treatment group, a single subcutaneous dose of PEG-rhG-CSF was administered 24 hours after completion of oxaliplatin chemotherapy on day 1. Dosage was weight-based: 6 mg for patients weighing ≥45 kg and 3 mg for those weighing <45 kg. The control group did not receive primary prophylactic PEG-rhG-CSF therapy.

In each cycle, day 1 was designated as the start date, with complete blood counts (CBCs) performed on days 0, 7, and 15; additionally, clinically indicated CBCs (eg, for fever or suspected infection) were obtained and included in determining the ANC nadir for each cycle. Adverse events were recorded, along with the administration of rescue recombinant human granulocyte colony-stimulating factor (rhG−CSF)—initiated when ANC fell below 1.0 × 10^9^/L, using subcutaneous injections at 5 µg/kg/day until ANC recovered to >2 × 10^9^/L—and the use or non−use of antibiotics.

### 2.3 Study endpoints and assessments

The primary endpoint was safety, with all adverse events (AEs) graded and documented according to the National Cancer Institute’s Common Terminology Criteria for Adverse Events, version 5.0 (CTCAE 5.0).

Secondary endpoints included the incidence of grade ≥2 CIN, the incidence of FN, ANC levels at each time point, and CIN-related complications such as chemotherapy delays, dose reductions, and the need for rescue therapy. Chemotherapy delay was defined as postponement of treatment by ≥3 days, while dose reduction was defined as an actual chemotherapy dose ≤80% of the planned dose. CIN was graded as follows: grade 1, ANC (<2.0–1.5) × 10^9^/L; grade 2, ANC (<1.5–1.0) × 10^9^/L; grade 3, ANC (0.5–<1.0) × 10^9^/L; and grade 4, ANC <0.5 × 10^9^/L. FN was defined as severe neutropenia, namely ANC <0.5 × 10^9^/L, or ANC <1.0 × 10^9^/L with a predicted decline to <0.5 × 10^9^/L within 48 hours, accompanied by fever—oral temperature ≥38.3 °C, or >38.0 °C persisting for more than one hour.

### 2.4 Statistical analysis

Data were analyzed using SPSS version 27.0, and figures were generated with Origin 2022. For normally distributed continuous variables, means were compared between groups using the t-test and presented as mean ± standard deviation. Non-normally distributed data were analyzed using the nonparametric rank-sum test. Categorical variables were compared using the chi-square test or Fisher’s exact test, with results expressed as frequencies (percentages). Ordinal categorical variables were assessed using the Mann–Whitney U test. All statistical tests were two-sided, with a P-value <0.05 considered statistically significant.

## 3 Results

### 3.1 Patient baseline characteristics

**Figure 1:**
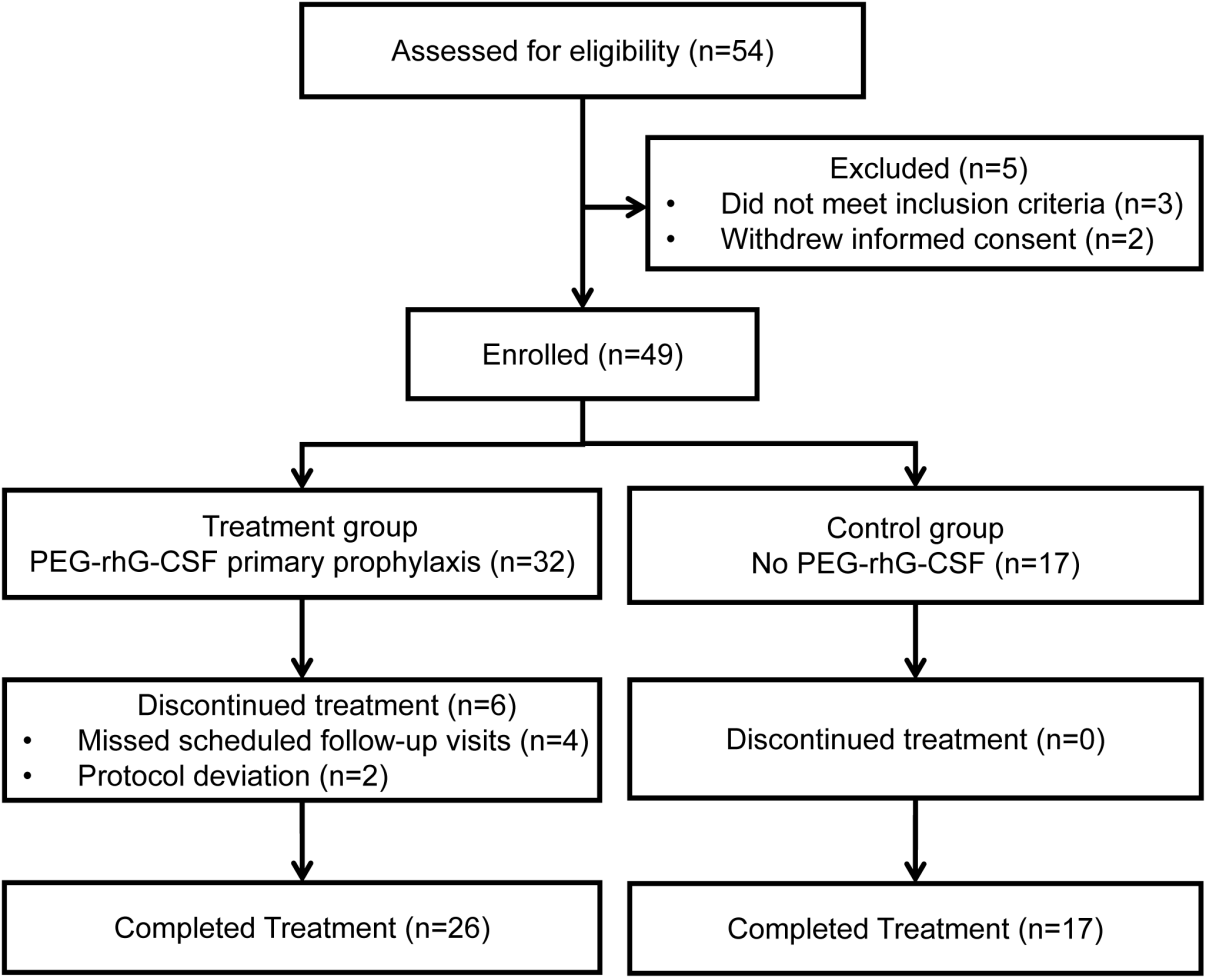
Patient enrollment and disposition. PEG-rhG-CSF, pegylated recombinant human granulocyte colony-stimulating factor.

Between March 2022 and January 2023, a total of 54 patients were screened, of whom 49 met the eligibility criteria—32 in the treatment group and 17 in the control group. Ultimately, 43 patients—26 in the treatment group and 17 in the control group—completed two consecutive chemotherapy cycles and were included in the analysis. Six patients were excluded due to failure to complete the scheduled hematologic monitoring for personal reasons. In total, data from 86 chemotherapy cycles were collected—52 cycles in the treatment group and 34 in the control group (**Figure 1**).

All patients in the treatment group weighed ≥45 kg and therefore received 6 mg of PEG-rhG-CSF. The mean patient age was 61.3 years; 43 patients (87.8%) were male, 42 (85.7%) had gastric cancer, and 35 (81.4%) received the SOX regimen. No statistically significant differences (*p* > 0.05) were observed between the two groups in terms of age, sex, disease stage, tumor type, chemotherapy regimen, or baseline white blood cell count (WBC) and ANC levels (**Table 1**).

**Table 1:** Baseline demographics and clinical characteristics of patients in the treatment and control groups.

Characteristic	Total (n = 49)	Treatment group (n = 32)	Control group (n = 17)	*P*
Age (years), mean ± SD	61.3 ± 4.8	62.5 ± 7.9	59.0 ± 6.9	0.126
Sex, n (%)				0.650
Male	43 (87.8%)	27 (84.4%)	16 (94.1%)	
Female	6 (12.2%)	5 (15.6%)	1 (5.8%)	
BMI (kg/m²), mean ± SD	21.3 ± 2.8	21.0 ± 2.7	21.8 ± 3.0	0.343
Body surface area (m²), mean ± SD	1.66 ± 0.15	1.65 ± 0.16	1.67 ± 0.14	0.631
TNM stage*, n (%)				0.498
II	12 (24.5%)	6 (18.8%)	6 (35.3%)	
III	28 (57.1%)	20 (62.5%)	8 (47.1%)	
IV	9 (18.4%)	6 (18.8%)	3 (17.7%)	
Tumor type, n (%)				0.217
Gastric cancer	42 (85.7%)	29 (90.6%)	13 (76.5%)	
Colorectal cancer	7 (14.3%)	3 (9.4)	4 (23.5%)	
Chemotherapy regimen, n (%)				0.106
SOX	35 (81.4%)	29 (90.6%)	12 (70.6%)	
CAPOX	8 (18.6%)	3 (9.4%)	5 (29.4%)	
History of chemotherapy, n (%)				0.604
Yes	34 (67.4%)	23 (72.0%)	11 (64.7%)	
No	15 (32.6%)	9 (28.1%)	6 (35.3%)	
ECOG PS, n (%)				0.769
0	24 (51.2%)	15 (47.0%)	9 (52.9%)	
1	25 (48.8%)	17 (53.1%)	8 (47.1%)	
WBC (×10^9^/L), median (IQR)	4.90 (3.71–5.81)	4.67 (3.51–7.38)	4.49 (3.97–5.27)	0.801
ANC (×10^9^/L), median (IQR)	2.46 (1.97–5.60)	2.55 (1.82–4.84)	2.39 (2.19–3.42)	0.908

BMI Body Mass Index, SOX S-1 plus oxaliplatin, CAPOX capecitabine plus oxaliplatin, ECOG PS Eastern Cooperative Oncology Group performance status, WBC white blood cell count, ANC absolute neutrophil count.

*TNM stage based on the American Joint Committee on Cancer (AJCC) Staging System, 8th Edition.

### 3.2 Safety

**Figure 2:**
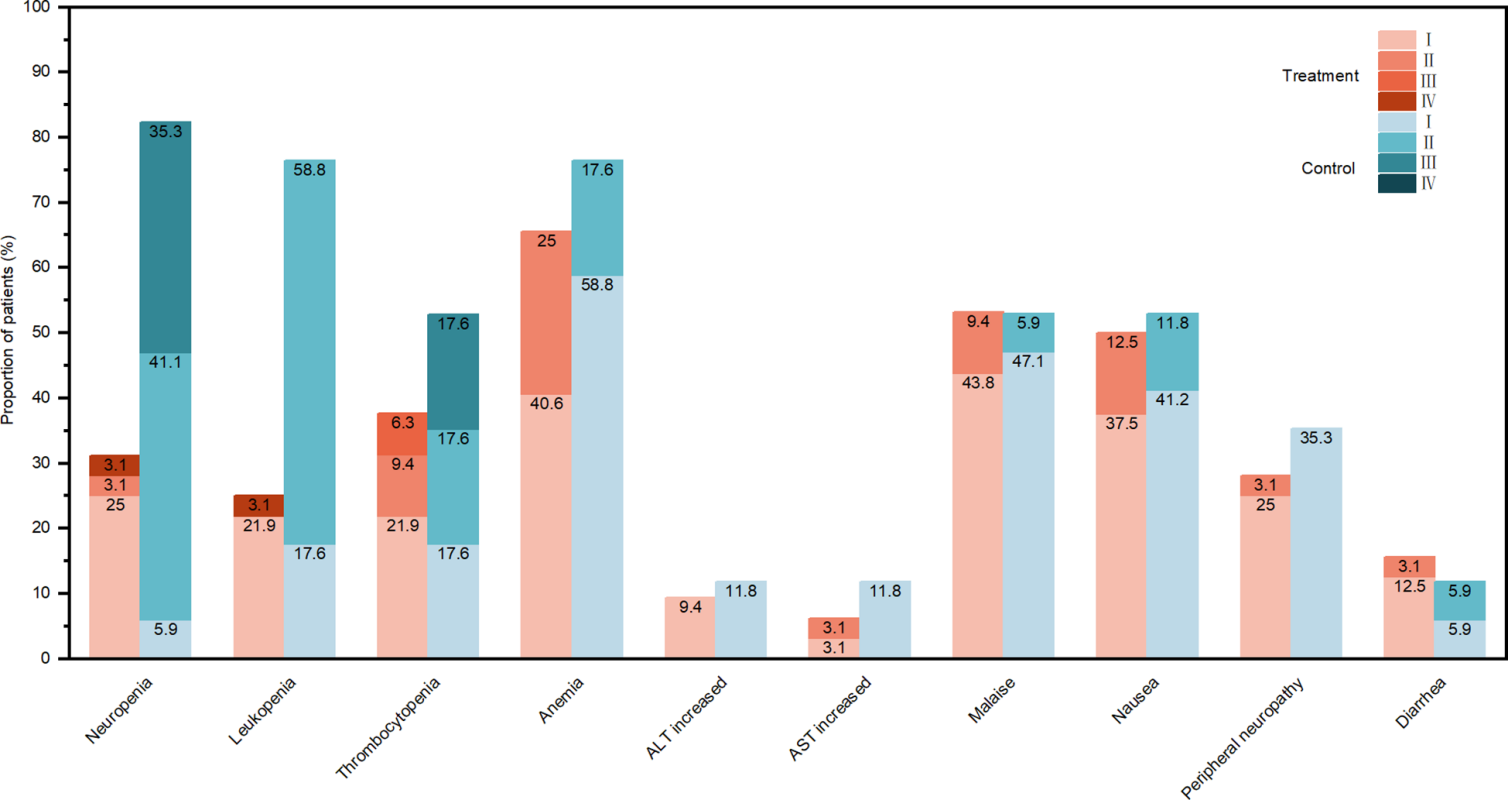
Incidence of all-grade adverse events (AEs). ALT, alanine aminotransferase; AST, aspartate aminotransferase.

The overall incidence of AEs was 93.8% in the treatment group and 100.0% in the control group, with no statistically significant difference (*p* = 0.542). Hematologic toxicity was the most common adverse event. Among all-grade hematologic toxicities, the incidences of leukopenia and neutropenia were significantly lower in the experimental group than in the control group (*p* < 0.001), whereas the rates of anemia and thrombocytopenia showed no significant differences. For grade ≥3 hematologic toxicities, the incidence of neutropenia was markedly lower in the experimental group (3.1% vs. 35.3%, *p* = 0.005). No statistically significant differences were observed between groups in the incidence of grade ≥3 thrombocytopenia (6.3% vs. 17.6%, *p* = 0.326) or leukopenia (3.1% vs. 0, *p* = 1.000). No significant differences were observed between the two groups in the incidence of common non-hematologic toxicities, such as fatigue and nausea (**Figure 2**).

AEs definitively attributed to PEG-rhG-CSF in the treatment group included musculoskeletal pain and injection-site pain. Musculoskeletal pain was observed in five patients (15.6%), predominantly mild and transient in nature. Injection-site pain occurred in six patients (18.8%), all of which resolved spontaneously (**Supplementary Table 1**).

### 3.3 Efficacy

#### 3.3.1 The incidence of grade ≥ 2 CIN

Analysis was conducted according to the intention-to-treat (ITT) principle, with all six dropouts in the experimental group counted as having developed grade ≥2 neutropenia. Results showed that the incidence of grade ≥2 CIN was 25.0% (8/32) in the treatment group compared with 76.5% (13/17) in the control group, a statistically significant difference (*p* < 0.001). Additionally, a sensitivity analysis was performed on the per-protocol population (PPP). After excluding the six dropouts from the treatment group, the incidence of grade ≥2 CIN was 7.7% (2/26), with the difference between groups remaining significant (*p* < 0.001).

**Figure 3:**
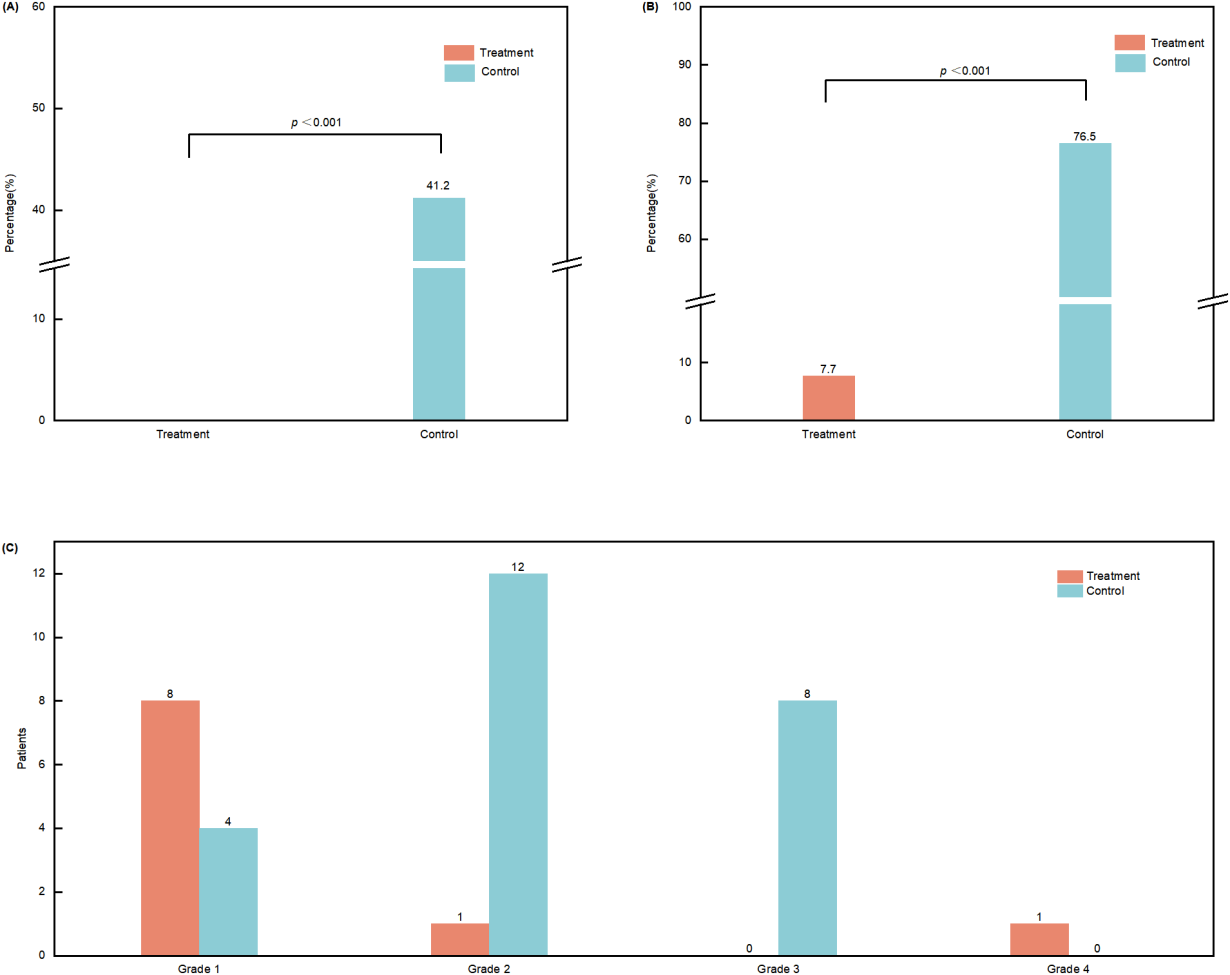
Incidence and severity of chemotherapy−induced neutropenia (CIN). **(A)** Incidence of grade ≥ 2 CIN in cycle 1. **(B)** Incidence of grade ≥ 2 CIN in cycle 2. **(C)** Distribution of CIN severity by grade. CIN severity was classified according to the Common Terminology Criteria for Adverse Events (CTCAE), version 5.0.

Across all 86 chemotherapy cycles, the incidence of grade ≥2 CIN was 3.8% (2/52) in the treatment group, significantly lower than 58.8% (20/34) in the control group (*p* < 0.001). This difference remained statistically significant in both cycle 1 (0 vs. 41.2%) and cycle 2 (7.7% vs. 76.5%) (*p* < 0.001) (**Figures 3A, B**).

Regimen-specific analysis revealed that among patients receiving the SOX regimen (S-1), the incidence of grade ≥2 CIN was 4.3% in the treatment group, significantly lower than 62.5% in the control group (*p* < 0.001). Similarly, among those treated with the CAPOX regimen (capecitabine), the incidence was 0 in the treatment group, markedly lower than 50.0% in the control group (*p* = 0.032).

#### 3.3.2 Distribution of CIN grades

The treatment group demonstrated generally milder CIN, with grade 1 CIN observed in 15.4% (8/52) of cycles. In contrast, the control group exhibited substantially greater severity, with CIN of grade ≥ 2 occurring in 58.8% (20/34) of cycles. The distribution of CIN grades differed significantly between the two groups (p < 0.001) (**Figure 3C**).

#### 3.3.3 Distribution of ANC

**Figure 4:**
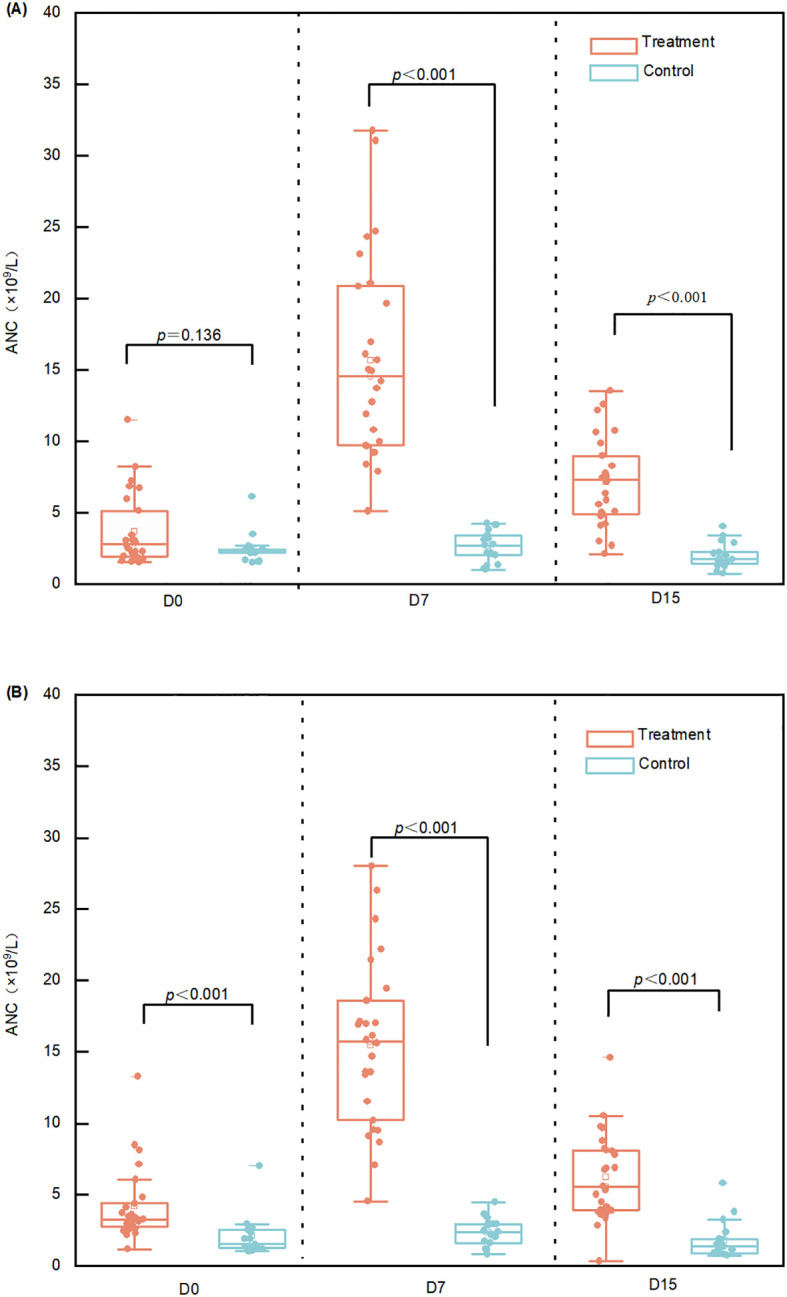
Distribution of absolute neutrophil counts (ANC). **(A)** Distribution of ANC in cycle 1. **(B)** Distribution of ANC in cycle 2. Dots in the scatter and box plots represent ANC measured on days 0, 7, and 15 of each cycle in both groups. ANC, absolute neutrophil count.

In both the first and second treatment cycles, the treatment group exhibited significantly higher ANC levels than the control group at all post-treatment sampling points (day 7 and day 15) (*p* < 0.001), excluding baseline measurements (**Figure 4**).

#### 3.3.4 Incidence of FN

During the study period, one patient in the treatment group (3.8%) developed FN, while no cases occurred in the control group, a difference that was not statistically significant (*p* = 1.000). The affected patient recovered following treatment with antibiotics and rescue rhG-CSF.

#### 3.3.5 CIN−related complications

**Figure 5:**
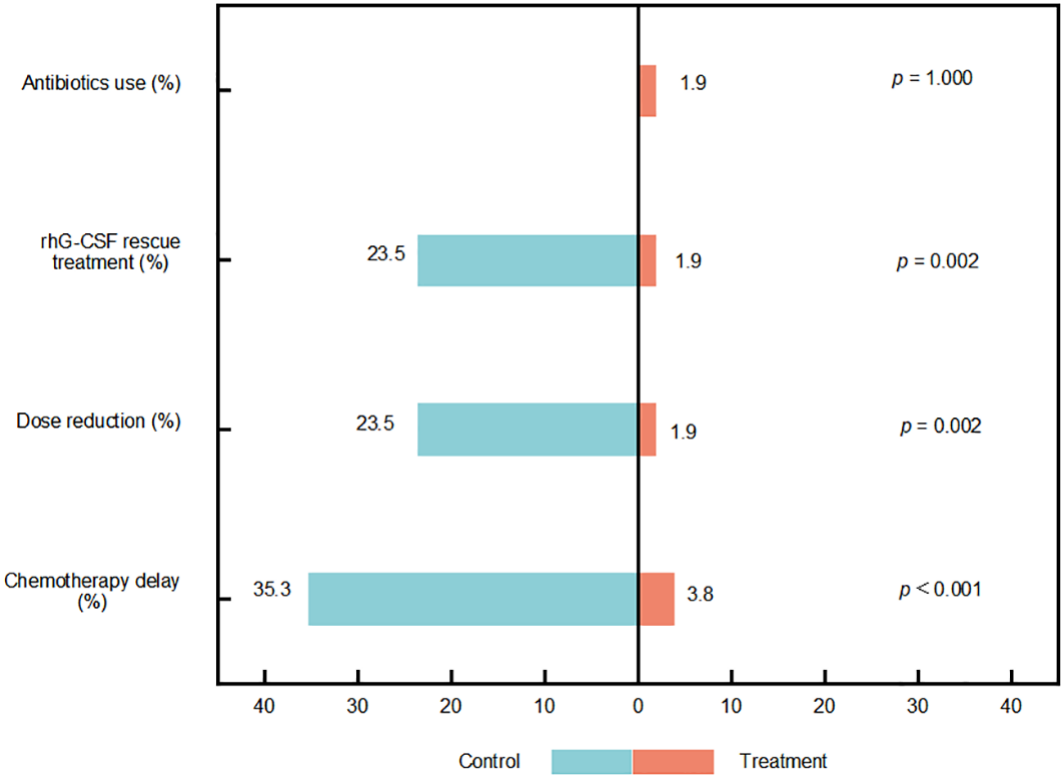
Incidence of chemotherapy−induced neutropenia (CIN)−related complications. rhG−CSF, recombinant human granulocyte colony−stimulating factor.

Compared with the control group, the treatment group demonstrated markedly lower rates of chemotherapy delays due to CIN (3.8% vs. 35.3%, *p* < 0.001), dose reductions (1.9% vs. 23.5%, *p* = 0.002), and rescue rhG-CSF use (1.9% vs. 23.5%, *p* = 0.002) (**Figure 5**).

## 4 Discussion

This prospective study evaluated the value of primary prophylactic PEG-rhG-CSF in patients with gastrointestinal malignancies undergoing regimens containing oral chemotherapeutic agents. The findings demonstrate that this strategy is not only well-tolerated without adding hematologic toxicity but also markedly reduces the incidence of clinically significant grade ≥2 CIN and its associated complications.

PEG-rhG-CSF, a member of the granulocyte colony-stimulating factor (G-CSF) family, is engineered by covalently conjugating polyethylene glycol (PEG) to its N-terminus, thereby prolonging its half-life and reducing dosing frequency (20). According to prescribing information and clinical guidelines, its administration is not recommended within 14 days prior to cytotoxic chemotherapy or within 24 hours after treatment (21). This timing restriction is based on the theoretical premise that G-CSFs drive chemotherapy-sensitive myeloid progenitors into active proliferation, and overlapping exposure may exacerbate myelosuppression and hematologic toxicity. However, an increasing body of evidence is beginning to challenge this long-standing assumption.

A study in breast cancer patients comparing same-day versus next-day administration of pegfilgrastim (PEG-rhG-CSF) reported a significantly higher FN incidence in the same-day group (24.6% vs. 11.7%, *p* = 0.002), suggesting that same-day dosing may impose an additional burden of myelotoxicity (22); In contrast, no such increase in myelotoxicity was observed in lung cancer cohorts, implying that the chemotherapy backbone may be a critical modulating factor (23). According to the 2022 NCCN Guidelines for Hematopoietic Growth Factors, the dose-dense doxorubicin/cyclophosphamide (ddAC) regimen used in breast cancer carries a high FN risk, whereas most non–small cell lung cancer regimens are generally low risk. Further stratified meta-analyses corroborate that, in low-FN-risk regimens and specific tumor types, same-day pegfilgrastim does not increase FN incidence or myelotoxicity, aligning with the risk-stratification hypothesis (24). In our study, the SOX and CAPOX regimens employed were not high-risk, and the observed safety profile was consistent with the aforementioned evidence.

It is worth emphasizing that clinical evidence has yet to directly substantiate the longstanding hypothesis that “G−CSF–induced proliferating myeloid cells are more vulnerable to chemotherapy-induced cytotoxicity.” In contrast, animal studies by Petros et al revealed that combining pegfilgrastim with 5−fluorouracil alleviated both bone marrow suppression and peripheral granulocytopenia without amplifying myelotoxicity, thereby offering mechanistic support for same-day or near-concurrent administration in the setting of low- to moderate-risk chemotherapy (25). Collectively, these findings suggest that, in intermediate-FN-risk oral fluoropyrimidine–based regimens such as SOX and CAPOX, same-day or near-term dosing of PEG−rhG−CSF is unlikely to heighten myelotoxicity; however, in high-risk regimens such as ddAC, adherence to the ≥24−hour post-chemotherapy interval remains essential to minimize the potential for additive toxicity.

For patients receiving prolonged oral chemotherapy regimens such as capecitabine or S−1, which require continuous administration for 14 days, even grade 2 CIN can precipitate treatment interruption, thereby compromising therapeutic efficacy. Our study demonstrates that primary prophylaxis with PEG−rhG−CSF effectively reduced the incidence of grade ≥2 CIN from 58.8% to 3.8%, while significantly decreasing chemotherapy delays and dose reductions attributable to neutropenia. This finding offers a pivotal strategy for safeguarding treatment continuity and dose intensity in this patient population. Stratified analysis by chemotherapeutic agent revealed that, compared with capecitabine, S−1 combined with PEG−rhG−CSF conferred a more pronounced reduction in the incidence of grade ≥2 CIN. This discrepancy is likely attributable to inherent differences in the myelosuppressive profiles of these two oral fluoropyrimidines. Our data indicate that, regardless of PEG−rhG−CSF use, the incidence of leukopenia and neutropenia was consistently higher in the S−1 group than in the capecitabine group, suggesting a higher baseline granulocytic toxicity with S−1 and a correspondingly greater benefit from growth factor prophylaxis. These findings align with prior evidence. A randomized controlled trial (26) in perioperative gastric cancer treatment reported that the SOX regimen induced grade ≥2 neutropenia in 14.0% of patients, significantly higher than the 6.0% observed with CAPOX, consistent with our results. Taken together, the evidence favors prioritizing PEG−rhG−CSF prophylaxis in regimens containing S−1 to mitigate CIN risk. Nonetheless, caution is warranted, as the number of capecitabine cases in our study was relatively small, introducing the potential for selection bias and unstable estimates. Larger prospective studies are needed to confirm these observations.

As an exploratory, nonrandomized study, several limitations should be acknowledged. First, despite balanced baseline characteristics, selection bias and unmeasured confounding cannot be excluded. Second, no formal sample−size calculation was performed and the sample size was modest, limiting statistical power—particularly for rare outcomes such as febrile FN. In addition, analyses across multiple endpoints, subgroups, and time points were not adjusted for multiplicity, increasing the risk of type I error. Third, during the COVID−19 pandemic, reduced complete blood count (CBC) sampling may have limited the precise ascertainment of ANC nadirs.

We will endeavor to expand the sample size and, in future studies, adopt a randomized design, increase the frequency of blood sampling, and incorporate multiple dosing cohorts, with the aim of generating higher−level evidence to guide the use of PEG−rhG−CSF in such specialized chemotherapy regimens.

## 5 Conclusions

For patients with GI cancer receiving oral chemotherapy regimens such as capecitabine or S−1, primary prophylaxis with PEG−rhG−CSF appeared to be well tolerated and was associated with a reduced incidence of clinically meaningful grade ≥2 CIN and CIN-related complications, without evidence of increased hematologic toxicity. Given the exploratory, nonrandomized design and limited sample size, these findings should be considered hypothesis-generating and warrant confirmation in larger randomized trials.

## Data Availability

The original contributions presented in the study are included in the article/**Supplementary Material**. Further inquiries can be directed to the corresponding authors.
